# AQP1 in the Gastrointestinal Tract of Mice: Expression Pattern and Impact of AQP1 Knockout on Colonic Function

**DOI:** 10.3390/ijms24043616

**Published:** 2023-02-10

**Authors:** Stefanie Volkart, Urs Kym, Olivier Braissant, Edgar Delgado-Eckert, Samer Al-Samir, Rebecca Angresius, Zihe Huo, Stefan Holland-Cunz, Stephanie J. Gros

**Affiliations:** 1 Department of Pediatric Surgery, University Children’s Hospital Basel, 4056 Basel, Switzerland; 2 Department of Clinical Research, University of Basel, 4001 Basel, Switzerland; 3 Microcalorimetry Unit, Department of Biomedical Engineering, University of Basel, 4001 Basel, Switzerland; 4 Computational Physiology and Biostatistics, Department of Biomedical Engineering at University of Basel and University Children’s Hospital Basel, 4056 Basel, Switzerland; 5 Vegetative Physiologie 4220, Zentrum Physiologie, Medizinische Hochschule Hannover, 30625 Hannover, Germany

**Keywords:** Aquaporin 1, hypoxia, intestinal oxygen gradient, enteric nervous system, myenteric plexus, submucosal plexus

## Abstract

Aquaporin 1 (AQP1) is one of thirteen known mammalian aquaporins. Its main function is the transport of water across cell membranes. Lately, a role of AQP has been attributed to other physiological and pathological functions including cell migration and peripheral pain perception. AQP1 has been found in several parts of the enteric nervous system, e.g., in the rat ileum and in the ovine duodenum. Its function in the intestine appears to be multifaceted and is still not completely understood. The aim of the study was to analyze the distribution and localization of AQP1 in the entire intestinal tract of mice. AQP1 expression was correlated with the hypoxic expression profile of the various intestinal segments, intestinal wall thickness and edema, as well as other aspects of colon function including the ability of mice to concentrate stools and their microbiome composition. AQP1 was found in a specific pattern in the serosa, the mucosa, and the enteric nervous system throughout the gastrointestinal tract. The highest amount of AQP1 in the gastrointestinal tract was found in the small intestine. AQP1 expression correlated with the expression profiles of hypoxia-dependent proteins such as HIF-1α and PGK1. Loss of AQP1 through knockout of AQP1 in these mice led to a reduced amount of bacteroidetes and firmicutes but an increased amount of the rest of the phyla, especially deferribacteres, proteobacteria, and verrucomicrobia. Although AQP-KO mice retained gastrointestinal function, distinct changes regarding the anatomy of the intestinal wall including intestinal wall thickness and edema were observed. Loss of AQP1 might interfere with the ability of the mice to concentrate their stool and it is associated with a significantly different composition of the of the bacterial stool microbiome.

## 1. Introduction

Aquaporins (AQP) are a group of water channels that have been found in many organs and tissues in different species, including humans, animals, plants, and lower organisms [1,2,3]. Thirteen different isoforms (AQP0-12) have been discovered in mammals [1]. As the name implies, one of the main functions of aquaporins is to transport water through cell plasma membranes and another function is to additionally transport other substrates such as glycerol, urea, ammonia, and some ions. A gas channel function has also recently been suggested [4,5]. In these functions, aquaporins contribute to many physiological and pathological processes. For example, they are known for their role in cell migration, cell proliferation, neural activity, brain water balance, epidermal hydration, and ocular function [1,2,6].

AQP1 has foremost been found in the kidney and in the lungs; its primary and best described function is the transport of water [7,8,9,10,11]. Eleven of the thirteen known aquaporins have been described to be expressed in the stomach and the intestines of mammals (AQP1-11). The distribution and location of the different types varies among different species [12,13]. Aquaporin 1 (AQP1) has been found in the ileum, the jejunum, and the colon [14,15,16] of mice. In the human intestine, AQP1 has been described in the esophagus, the stomach, the duodenum, the jejunum, the ileum, and the colon [12,13,17,18,19]. Its function in the intestine appears to be diverse and is not completely understood. Three types of aquaporins (AQP1, AQP2, and AQP4) have been found in neurons or glial cells in the enteric nervous system [20]. AQP1 has been detected in the submucosal and the myenteric plexus of the enteric nervous system of the human esophagus as well as in glial cells of the pancreas [19]. A study in 2011 reported AQP1 expression in the myenteric plexus of the ovine duodenum [21]. A further study showed AQP1 expression in a subgroup of enteric nervous cells, in HuC/D positive neurons, mainly in the myenteric plexus, and some in the submucosal plexus of the enteric nervous system of rats [22]. In the buffalo, a study from 2015 reported the presence of AQP1 in the small intestine as well as in the colon; in this study, a co-localization with enteric neurons in the jejunum, the ileum, the cecum, and the colon was shown [23]. However, the function of AQP1 in the enteric nervous system remains unclear. AQP1 has been implicated in pathophysiological processes such as pain perception, inflammatory processes, diabetes, and hypoxic conditions [24,25,26].

The hypoxia-inducible factor 1 alpha (HIF-1α) is known as a key regulator of cellular response to hypoxia and, therefore, plays an important role in the regulation of oxygen homoeostasis. Under hypoxic conditions, HIF-1α is upregulated and activates cellular responses to low oxygen levels [27,28]. Recent studies suggest that AQP1 expression is upregulated by a HIF-1α increase as a result of hypoxic conditions [29,30,31,32,33]. On the other hand, the upregulation of HIF-1α in the lungs under hypoxic conditions was inhibited in AQP1-knockout mice [34], suggesting a feedback mechanism between HIF-1α and AQP1 expression. One of the well described enzymes that are upregulated by HIF-1α is phosphoglycerate kinase 1 (PGK1). This glycolytic enzyme is upregulated when HIF-1α [35,36] rises and thus can be used as a measure of hypoxia.

The intestinal microbiota have been increasingly investigated in the past years. It has been shown that many diseases present with a shift in the bacterial population of the intestinal tract, as the gut microbiota play an important role in drug metabolism, prevention of pathogenic bacteria, and immune system maturation [37,38]. In the human and in the mouse gut, most of the bacteria belong to the phyla firmicutes and bacteroidetes [39,40,41]. A study from 2020 in rats analyzed the impact of α-synuclein in Parkinson disease; in the ileum, the authors found a neuronal loss in the submucosal plexus and an increase in enteric glial cells in the myenteric plexus. Additionally, this study found an alteration in the composition of the intestinal microbiome [42]. Recent studies have shown a correlation between the gut microbiome composition and functional aspects of the enteric nervous system [43,44]. An influence of AQP1 expression on the microbiota has not been investigated.

Taken together, it appears that the current knowledge on the localization and function of AQP1 in the intestines and the enteric nervous system in mammals remains incomplete. Although the functional impact of AQP1 expression in the intestinal tracts remains unclear, it is possible that AQP1 may inflict on the neuronal function of the enteric nervous system, both under physiological and pathophysiological conditions.

This study analyzes the localization of AQP1 in the entire intestinal tract of mice. AQP1 expression correlated with the hypoxic expression profile of the various intestinal segments, as well as with aspects of colon function including the ability of mice to concentrate stools and their microbiome composition.

## 2. Results

In the first part of the study, we characterized the expression of AQP1 throughout the gastrointestinal tract, the correlating expression of AQP1-regulating and hypoxia-inducible factors, and the resulting changes of the anatomical structure of the intestinal wall in the AQP1-KO animals.

### 2.1. AQP1 Expression Profile in the Gastrointestinal Tract

**Figure 1:**
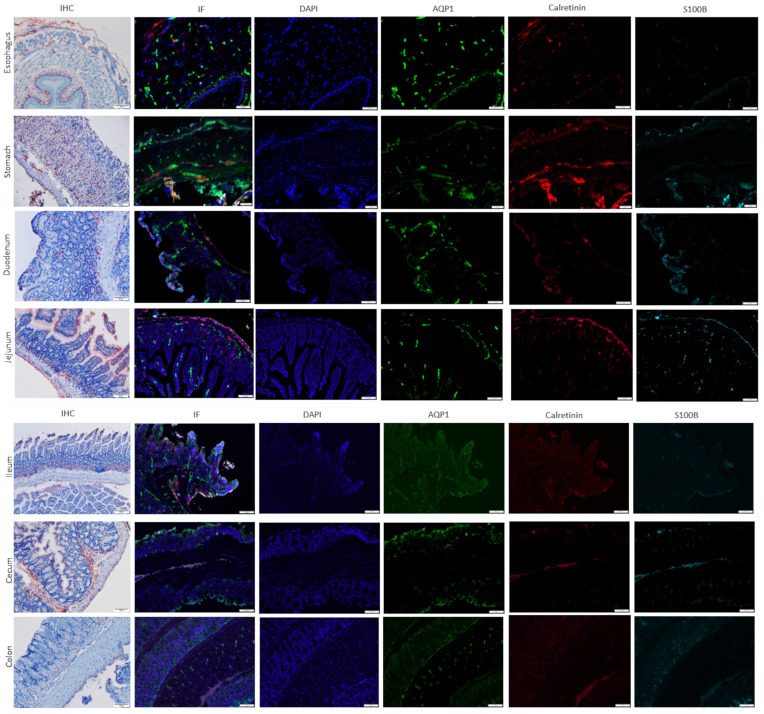

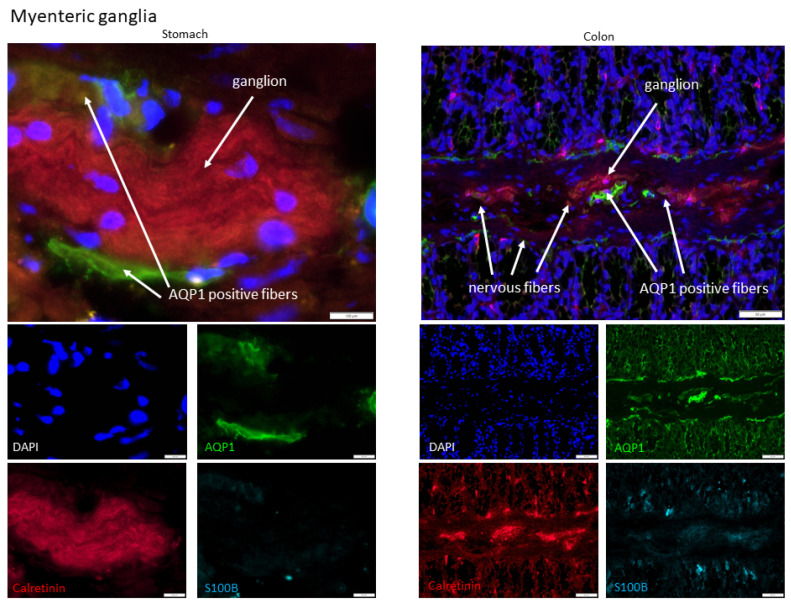
AQP1 expression in different parts of the gastrointestinal tract and co-localization with ganglia of the enteric nervous system. Figure 1 shows a representative picture from every segment of the gastrointestinal tract of WT mice. Immunohistochemistry showed an increased overall expression of AQP1 (red staining) in the small intestine particularly in the jejunum and ileum (scale bar 100 µm). Next to the serosa and submucosal glands in the esophagus, a high AQP1 expression was found in the mucosa. Depending on the segment, AQP1 was seen more prominently in the submucosal or myenteric plexus. The immunofluorescence staining confirmed the expression of AQP1 (green) (scale bar 100 µm). The calretinin positive ganglion was surrounded by AQP1 positive fibers in the absence of S100B positive glial cells in the stomach sample (scale bar 100 µm). In the colon sample there was a co-localization of APQ1, calretinin, and S100B in the nervous structures of the myenteric plexus (scale bar 50 µm). Nuclei were stained by DAPI (blue).

**Figure 2:**
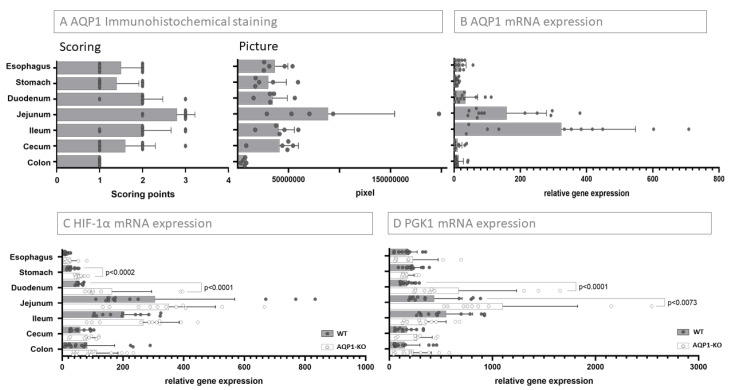
AQP1 expression and relation to hypoxia-related factors. (**A**) Scoring of AQP1 immunohistochemical staining and overall expression of AQP1 determined by immunohistochemical staining through scoring (left graph) and by integrated density picture analysis (right graph) in WT mice. In both graphs the highest amount of AQP1 was found in the small intestine (jejunum, followed by ileum). Both methods showed a similar pattern of AQP1 distribution. (**B**) AQP1 mRNA expression. The relative gene expression confirmed the high expression of AQP1 protein in the jejunum and ileum on the mRNA level. (**C**) HIF-1α mRNA expression and (**D**) PGK1 mRNA expression. The mRNA expression of the hypoxia-dependent genes HIF-1α and PGK1 was shown in WT and AQP1-KO mice. The highest amounts of HIF-1α and PGK1 were found in the small intestine corresponding to the AQP1 expression. In all organs the loss of APQ1 led to an increase in HIF-1α and PGK1 in the AQP1-KO compared to the WT mice. The increase in HIF-1α was significant in the duodenum (*p*-value < 0.0001) and the stomach (*p*-value < 0.0002). PGK1 was increased in the AQP1-KO mice compared to WT mice except for the stomach and ileum. The increase in PGK1 in the AQP1-KO compared with the WT mice was significant in the duodenum and the jejunum (*p*-value < 0.0001 and <0.007, respectively).

In order to characterize the distribution of AQP1 along the intestinal tract, we stained all segments of the intestinal tract by H.E. staining, by staining AQP1 immunohistochemically, and by co-staining AQP1, calretinin, and S100B by immunofluorescence staining. AQP1 has been proven to be positive in the endothelial cells of blood vessels, which we confirmed throughout and which served as an internal positive control in all stainings. We found AQP1 to be positive to some extent in the mucosa throughout all segments of the intestinal tract, although markedly more in the small intestine, as well as in the esophagus. AQP1 expression in the serosa layer was most prominent in the esophagus. Not surprisingly, the submucosal glands were highly positive for AQP1. Furthermore, AQP1 positivity was found primarily in the structures of the enteric nervous system. While the esophagus did not show much expression of AQP1 either in the myenteric or in the submucosal plexus, AQP1 expression was present in the submucosal plexus and the myenteric plexus in all other intestinal organs. However, the submucosal plexus was more prominent in the segments of the small intestine compared to the colon. In the enteric nervous system, AQP1 localizes around the calretinin positive ganglia and colocalizes with some S100B positive nervous fibers. Figure 1 shows a prepresentative immunohistochemical and immunofluorescence staining of each organ as well as the localization of AQP1 in relation to calretinin and S100B in an enteric ganglion (pictures of AQP1 expression of WT vs. AQP1-KO mice are shown in Supplementary Figure S1). These distinctly different locations of presentation might suggest a different role of AQP1 in each location. When comparing the overall expression of AQP1 including all locations of AQP1 expression, the highest amount of AQP1 in the gastrointestinal tract in the mouse was found in the jejunum, followed by the ileum and equally by the duodenum (Figure 2A). These two were closely followed by the cecum, then the esophagus and the stomach. The lowest AQP1 expression was seen in the colon. To objectify these results, we performed an integrated density analysis for AQP1. It confirmed the described pattern along the intestinal tract (Figure 2A). In detail, the most integrated density for AQP1 analyzed with a threshold of HSB by 195–255/30–255/0–200 was found in the jejunum with a mean of 88,655,936 px, followed by the cecum, the ileum, the esophagus, the duodenum, the stomach, and the colon (6,382,216 px).

For further confirmation, mRNA-analysis was performed for each segment and showed the highest relative expression of AQP1 mRNA to be in the ileum, followed by the jejunum and the duodenum. Less AQP1-mRNA was found in the esophagus, the colon, the cecum, and the stomach (Figure 2B). Overall, there was a statistically significantly higher AQP1 mRNA expression in the small intestine compared to the rest of the organs (*p*-value < 0.007). RNA expression of AQP1-KO mice was confirmed to be negative (Supplementary Figure S2).

### 2.2. Relation of AQP1 Expression to the Known Regulatory Factor HIF-1α

Based on the knowledge that AQP1 expression is regulated by HIF-1α expression, e.g., under hypoxic conditions, we examined HIF-1α mRNA expression in WT and AQP1-KO mice. HIF-1α mRNA analysis in the wildtype mice shows the highest amount of HIF-1α in the jejunum, followed by a declining amount of HIF-1α in the ileum, the colon, the cecum, the duodenum, the stomach, and the esophagus. The expression between the small intestine and the rest of the organs showed a significant elevation (*p* < 0.008). In all organs, the amount of HIF-1α in the AQP1-KO mice was higher than in the WT mice with a similar distribution pattern in the jejunum, ileum, duodenum, colon, cecum, stomach, and esophagus (Figure 2C). The differences between WT and AQP1-KO mice were only significant in the duodenum and the stomach (*p* < 0.0001 and < 0.0002).

We further investigated the mRNA expression pattern of PGK1, as its expression is also upregulated by HIF-1α and can indirectly give a further indication of HIF-1α regulation in the absence of AQP1. RNA analysis for PGK1 showed the highest level in the WT mice in the ileum followed by the jejunum, the stomach, the esophagus, the duodenum, and the cecum (Figure 2D). In accordance with the HIF-1α expression, the difference between the small intestine and the rest of the organs was significant (*p* < 0.02). In the AQP1-KO mice the amount of PGK1 was, with the exception of the stomach and the ileum, higher than in the WT mice. This increase was significant in the duodenum and the jejunum (*p* < 0.0001 and < 0.007).

### 2.3. Characterization of the Intestinal Wall Thickness

**Figure 3:**
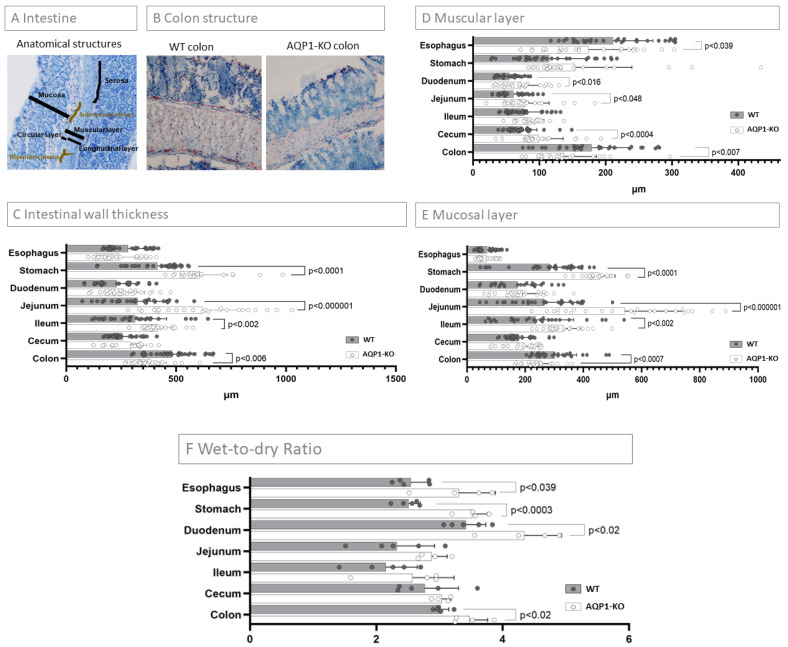
WT versus AQP1 Knockout: Analysis of the intestinal wall. (**A**) Intestine: Anatomical stuctures. The intestinal wall is comprised, from outside to inside, of the serosa, the longitudinal muscular layer, the myenteric plexus, the circular muscular layer, the submucosa with the submucosal plexus, and the mucosa. The measurements of the intestinal wall thickness were taken according to the labelling in the figure. (**B**) Differences in colon structure, It already can be visually observed in the immunohistochemical staining (both 20× magnification), that there were obvious differences in the intestinal wall structure between AQP1-KO and WT mice. (**C**) Intestinal wall thickness. Depicted are the thickness measurements of the entire intestinal wall in WT and AQP1-KO mice. In the stomach, jejunum, and ileum the loss of AQP1 led to a significant increase in abdominal wall thickness (*p* < 0.0001, *p* < 0.00001, and *p*-value < 0.002, respectively), while, as seen in B above, it can be confirmed that the intestinal wall thickness decreased significantly in the colon of AQP1-KO compared to WT mice (*p* < 0.006). (**D**) Muscular layer/(**E**) mucosal layer in (**D**,**E**) the thickness of the muscular layer and the mucosal layer of the abdominal wall are depicted for all intestinal segments separately. In both mucosal and muscular layers, the trends were similar to the measurements of the entire wall. However, a major increase in the mucosal layer was noted in stomach, jejunum, and ileum (*p* > 0.001, *p* < 0.000001, and *p* < 0.002, respectively), while both the mucosal and muscular layer decreased in the colon of AQP1-KO compared to WT mice (*p *< 0.0007 and *p <* 0.007, respectively). (**F**) Wet-to-dry ratio. The wet-to-dry ratio was determined by calculating: (wet weight–dry weight)/dry weight. An increase in intramural water content was observed in all segments.

A remarkable difference in the intestinal wall thickness was visually obvious between WT and AQP1-KO mice, especially in the colon, and therefore characterized in more detail (Figure 3B). Measurements of the entire intestinal wall, as well as the muscular and mucosal layers separately, were taken from the staining pictures (exemplarily shown in Figure 3A). The measurements for both WT and AQP1-KO mice are listed in Table S1 and depicted in Figure 3C. When comparing WT to AQP1-KO mice the intestinal wall of the AQP1-KO mice was thicker in the stomach (*p* < 0.0001), the duodenum, the jejunum (*p* < 0.00001), the ileum (*p* < 0.002), and the cecum. However, in the esophagus and the colon (*p* < 0.006), the intestinal wall AQP1-KO was thinner compared to the WT mice.

The measurements for the muscular layer are summarized in Table S2 and depicted in Figure 3D. The muscular layer of the AQP1-KO mice was thicker in the stomach, the duodenum, the jejunum, the ileum, and the cecum compared to the WT mice (Table S2 and Figure 3D). This difference was significant in the duodenum (*p* < 0.012), the jejunum (*p* < 0.049), and the cecum (*p* < 0.0004). In the esophagus (*p* < 0.039) and the colon (*p* < 0.007) the muscular layer was significantly thinner in the AQP1-KO compared to the WT mice.

With the exception of the esophagus, the mucosa represented the largest part of the intestinal wall in the gastrointestinal tract of WT and AQP1-KO mice (Table S3 and Figure 3E). The mucosal layer was thicker in the AQP1-KO compared to the WT mice in the stomach, the duodenum, the jejunum, the ileum, and the cecum. Notably, there was a significant increase in mucosal thickness in the jejunum (*p* < 0.000001) and the ileum (*p* < 0.002). The mucosal layer was thinner in the esophagus and the colon of AQP1-KO compared to the WT mice. This difference was significant in the colon (*p* < 0.0007).

### 2.4. Wet-To-Dry Ratio of Intestinal Segments

We observed an increase in the wet-to-dry ratio in all intestinal segments of the AQP1-KO mice, indicating an increased intramural water content in the intestine of AQP1-KO mice (Figure 3F). These changes were significant in all intestinal segments except for jejunum, ileum, and cecum.

In the first part of the study, we found three distinct locations of AQP1 expression in the serosa, the mucosa, and the enteric nervous system, each pointing to a different function of AQP1 in each anatomical aspect or segment. The presence of HIF-1α mRNA expression, as well as the mRNA expression of its downstream target PGK1, which were activated as key regulators in response to hypoxia, correlated with the AQP1 mRNA expression pattern in the intestinal segments. A similar pattern was also found for AQP1 protein expression in the immunostaining. Most interestingly, the anatomical changes that we could relate to the AQP1-KO, involved a significantly increased thickness of the mucosal layer in the parts of the small intestine with the highest physiological AQP1 expression and a significantly reduced thickness of the intestinal wall of the colon regarding the mucosal as well as the muscular layers. These findings, together with the expression of AQP1 in close proximity to ganglia and fibers of the enteric nervous system of submucous and myenteric plexus, suggest a functional consequence resulting from the lack of AQP1. Therefore, we investigated some aspects of colon function, including the ability of WT and AQP1-KO mice to concentrate stools, biophysical properties, and the microbiome of their stool.

### 2.5. Concentration of Stool

Dry weight of stools was measured for all WT and AQP1-KO mice. Although the results did not show significant differences between the groups, the ability to concentrate stools varied between the WT and AQP1-KO group. Overall, WT mice seemed to be more prone to liquidize their stool. This could be a sign that the passing of stool is normal or facilitated in WT mice, while AQP1-KO might have greater difficulty defecating. This could be a consequence of an impaired water homeostasis of the AQP1-KO mice, or it could have a more complex background.

### 2.6. Microbiome of Stool

**Figure 4:**
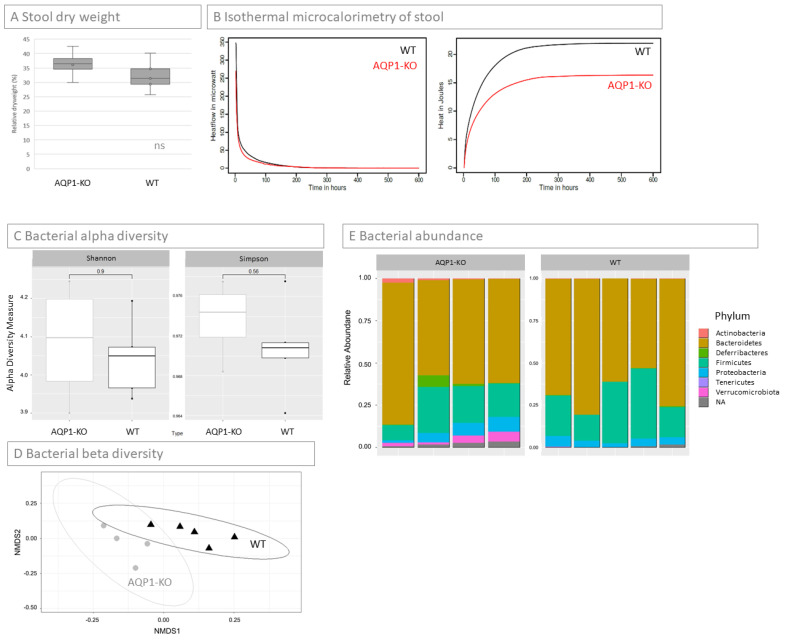
Stool analysis. (**A**) Dry weight of stool. While there were no significant differences in the dry weight of AQP1-KO (*n* = 4) and WT (*n* = 5) mice stools, it is notable that the standard deviation of AQP1-KO mice was much higher than that of WT mice (ns: not significant). (**B**) Heat release of stool. Isothermal microcalorimetry of pooled mouse stools revealed a reduced heat release of AQP1-KO stools compared to that of WT over time (first graph), resulting in a higher overall metabolic activity of WT stool during the observed time period (second graph). This is a first hint at a changed composition of microbiota in the AQP1-KO mice compared to the WT mice. (**C**) Bacterial alpha diversity. Alpha diversity in the AQP1-KO (*n* = 4) and WT (*n* = 5) group were measured using the Shannon and the Simpson indices, respectively. In both indices the groups were not significantly different. (**D**) Bacterial beta diversity. Bray–Curtis dissimilarities among the samples after the pairwise dissimilarities were transformed using non-metric multidimensional scaling (NMDS) in order to display the outcomes in two-dimensions. The differences between the two groups (WT and AQP1-KO) were assessed using non-parametric multivariate analysis of variance showing in a significant difference (*p* = 0.013). (**E**) Bacterial abundance. Relative abundance of the single phyla are shown from AQP1-KO (left) and WT (right) mice. The higher abundance of verrucomicrobia in the AQP1-KO compared to the WT was significant. Bacteroidetes and Firmicutes both have a slightly lower abundance in the AQP1-KO. (ns = not significant).

In order to test if the lack of AQP1 in the intestine resulted in changes of the microbiome, the stool of all mice was pooled in groups of WT and AQP1-KO mice and isothermal microcalorimetry was performed. A distinct difference in heat profile of the stool was observed with the AQP1-KO mice showing a decreased heat production rate and decreased total heat (Figure 4B). Metabolic heat production rate decreased in both cases following an exponential decay pattern, as the total heat produced over time showed a logarithmic pattern in both types of samples. However, the WT stool exhibited a higher metabolic heat production rate at the very beginning and activity was prolonged longer than for its AQP1-KO counterpart (Figure 4B first graph). Overall, this resulted in a higher total heat produced by the wild type for the same weight of stool (Figure 4B second graph). In order to characterize these changes, a microbiome analysis of the individual stool samples of each mouse was performed separately. The biodiversity was analyzed using alpha and beta diversity. Alpha-diversity showed little difference in the Shannon- and Simpson index when the WT with the AQP1-KO mice were compared (Figure 4C). The compositional dissimilarity of the samples was assessed using the Bray–Curtis statistic. Figure 4D depicts the Bray–Curtis dissimilarities among the samples after the pairwise dissimilarities were transformed using non-metric multidimensional scaling (NMDS) in order to display the outcomes in two-dimensions. The significance of this finding was assessed using non-parametric multivariate analysis of variance resulting in a significant *p*-value of 0.013.

To analyze the differences between bacterial composition in the WT and AQP1-KO mice, the microbiome was further investigated on the phylum level (Figure 4E). The relative abundance in the AQP1-KO mice compared to the WT growth in the phylum of the actinobacteria (0.13% WT, 1.09% AQP1-KO), the deferribacteres (0.21% WT, 2.32% AQP1-KO), the proteobacteria (4.29% WT, 5.85% AQP1-KO), and the verrucomicrobia phylum (0.03% WT, 3.43% AQP1-KO). The difference in the verrucomicrobia was significant (*p *< 0.009). The relative abundance in the bacteroidetes (67.94% WT, 66.1% AQP1-KO) and the firmicutes (26.92% WT, 19.39% AQP1-KO) were lower in the AQP1-KO compared to the WT mice. Overall, the absolute abundance in the AQP1-KO was higher (46,681 ± 7805) than that of the WT mice (38,775 ± 3576).

In the second part we show that the loss of AQP1 in the intestinal tract resulted in changes of anatomical structure of the intestinal wall, which might decrease the ability of the colon to concentrate stool and result in distinct changes of the microbiome.

## 3. Discussion

AQP1 expression was prominent in three distinct locations within the gastrointestinal tract of mice, in the serosa, the mucosa, and the enteric nervous system. Each location pointed towards a different possible function of AQP1 in each anatomical aspect or segment. Overall, AQP1 was found in all parts of the gastrointestinal tract but it was more prominent in some regions in certain locations than in other.

### 3.1. Role of AQP1 in the Gastrointestinal Tract Regarding Its Location

Expression of AQP1 in the serosa was most prominent in the esophagus, the only gastrointestinal organ that is not covered by a peritoneal sheath. One reason for a prominent AQP1 expression in this part could be an enhanced necessity for AQP1 in the esophageal to act towards water homeostasis. The presumed main function of AQP1 here would be that of a water channel. The expression of AQP1 in the mucosal layer presumably mainly fulfills a water channel function.

**Figure 5:**
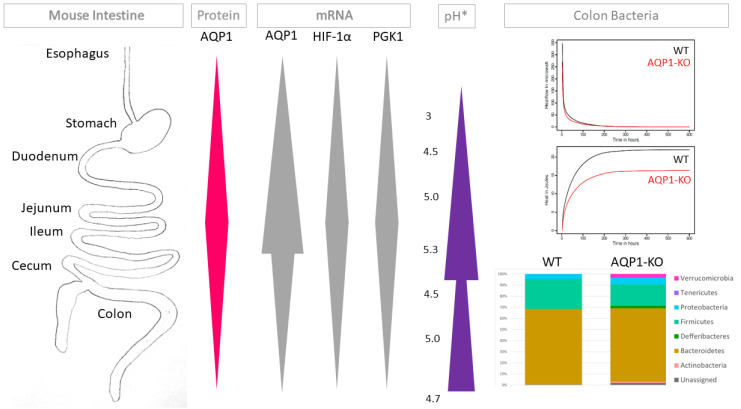
Summary of findings and context.

In the jejunum and ileum, the expression of AQP1 in the mucosa was especially high (Figure 5). This could particularly explain the increased transport function of AQP1 in jejunum and ileum, where water is an important component in dilution and resorption of nutrients and byproducts. Findings from a study of Sakai et al. from 2013 showed that gene expression of AQP1 in mice decreased from the jejunum down to the distal colon [14]. It has furthermore been suggested that one of the main functions of AQP1 in the intestine is to transport water between micro-vessels in the mucosa and the mucosa itself [12,45,46].

The third location, the expression of AQP1 in the cells of the enteric nervous system, was observed throughout the gastrointestinal tract. AQP1 was expressed in the submucosal as well as in the myenteric plexus of the enteric nervous system, suggesting a mechanism by which AQP1 contributes to neuronal function.

Throughout the gastrointestinal tract, the expression of AQP1 mRNA also correlated with the mRNA expression of the hypoxia-dependent factors HIF-1α and PGK1 (Figure 5). A similar pattern was also found for AQP1 protein expression. AQP1 expression, on the one hand, is known to be regulated by HIF-1α for example in tumors and might well be in the intestinal tract as well [29]. One indicator for this could be a compensatory increase in HIF-1α in the absence of AQP1 in the AQP1-KO mice. Arguably, it has previously been described that AQP1-KO mice did not upregulate HIF-1α under hypoxic conditions, while Echevarría et al. also found upregulation of HIF-1α in AQP1-knockdown normoxic endothelial cells [31,32,33,34]. On the other hand, HIF-1α does not only have an influence on AQP1 expression, but also plays a major role in the regulation of intra- and extracellular pH through its downstream targets. The luminal pH of the gastrointestinal tract of humans as well as mice is important for its function. Although pH values in the gastrointestinal tract did not show the same massive changes in mice as in humans, the values still ranged from the lowest pH of 3 in the esophagus to the highest pH of 5.3 in the ileum (Figure 5) [47,48]. These changes in pH were due to mucosal acid production in the stomach and gall output amongst others. Changes in pH facilitated the digestion and resorption of nutrients and play a role in regulating the incidence and prevalence of microbiota in the gastrointestinal tract (Figure 5). Here, it could be hypothesized that the absence of AQP1 also influenced the pH by leading to an increase in HIF-1α. To further evaluate this hypothesis, detailed pH measurements of AQP1-KO are needed.

### 3.2. Influence of AQP1-KO in Gastrointestinal Structure

Loss of AQP1 in the gastrointestinal tract did not lead to a complete loss of gastrointestinal function. Mice did not show growth deficits or increased occurrence of gastrointestinal disease. However, it led to distinct changes regarding the anatomy of the intestinal wall.

Looking at the organs with the highest physiological AQP1 expression, jejunum and ileum, the loss of AQP1 led to a significant increase in the intestinal wall thickness. This suggests that here the main function of AQP1 could be to maintain water homeostasis. One reason for this could be a swelling of the mucosa that occurs in the absence of AQP1. Water could be retained within the intestinal wall leading to a thickening, especially of the mucosal layer of stomach, jejunum, and ileum, probably the segments with the highest water fluctuation. Another explanation could lie in a hypertrophy of the mucosa. In that case, mice would aim to regulate water homeostasis in the absence of AQP1 by an increasing mucosal cell number. It is likely to assume a combination of both.

In our wet-to-dry measurements, we observed an increase in the wet-to-dry ratio in AQP1-KO mice in all segments of the intestinal tract. This indicates a disturbance of volume regulation in the absence of AQP1 and is in line with results from a previous study on the lens epithelial water permeability in AQP1-KO mice. Here, an approximately 3% greater basal water content was found in the lens of AQP1-KO mice compared to that of WT mice [49]. It has been shown previously that in the intestinal tract an increase in the wet-to-dry ratio is a sign of edema [50,51,52]. We therefore can conclude from our data that the knockout of AQP1 in mice led to edema of the intestinal wall. It has also previously been demonstrated that edema in the intestine leads to a reduction in muscle contractility, e.g., after traumatic brain injury [50,51,52].

In the literature there are several references, mostly on traumatic ischemia models, linking increased AQP1 expression with edema. In a rodent testicular ischemia–reperfusion model the increase in AQP1 expression was closely linked to testicular edema after reperfusion [53]. Similar findings have been described for lung edema following lung injury or lung exposure to hypobaric pressure and hypoxia, as well as myocardial edema following cardiac bypass surgery or myocardial infarction [54,55,56,57].

In our samples we observed AQP1 expression in WT mice most prominently in the mucosa and in cells of the enteric nervous system. Lack of AQP1 in AQP1-KO mice might therefore not only lead to edema, but also to a disturbance of the enteric nervous system and, possibly as a consequence of both changes, to alterations of smooth muscular contractility.

If we look at the colon, in which coordinated muscle activity is most important for passing stools, we found a significant increase in water content in the AQP1-KO mice compared to the WT mice. Hypothesizing that the intestinal edema leads to a reduced contractility of the intestinal muscle, we congruently observed a thinning of the entire intestinal wall including muscle and mucosal layer. Thus, a correlation between intestinal wall edema and wall thickness could be observed. Reduced contractility of the muscle layers could lead to changes of the stool passage time in the colon and thus lead to changes in the stool microbiome.

### 3.3. AQP1 in the Colon

If we look at the colon in particular, we found anatomical changes that we could relate to the AQP1-KO. They included a significantly reduced thickness of the intestinal wall of the colon affecting both the mucosal as well as the muscular layers. As we saw AQP1 expression throughout the intestinal tract in the submucosal as well as in the myenteric plexus of enteric nervous system, there might be a mechanism in which AQP1 contributes to changes in neuronal function resulting in reduced muscular and mucosal thickness in the colon. It has been described that the nerve fibers of the myenteric plexus in particular control the muscle tone and contractions of the intestine and that the submucosal plexus has its main function in controlling the secretion of the mucosa [58]. Gao at al. described the localization of AQP1 to be in satellite cells, a type of glia cell surrounding the ganglia cells [19]. This is in congruence with our observation of calretinin-positive ganglion cells that are surrounded by AQP1 positive fibers and in the colon S100B-positive nervous of the myenteric plexus. A study from 2017 showed that glia cells in mice affect the secretomotor function in the colon but the authors did not find an impact on water permeability [59]. It could be hypothesized that AQP1 in the glia cells could have an impact on the activity of glia cells in the enteric nervous system. AQP1 has also been suggested to act as a sensor of water homeostasis in parts of the nervous system [60]. Our data showed that AQP1 was found in close proximity to ganglia and fibers of the enteric nervous system of submucous and myenteric plexus. A possible function of AQP1 in regulation of cell–cell adhesion could also be considered to play a role in this process [61]. Assessing the muscle function of each intestinal segment could provide further insight into functional aspects of AQP1-knockout. Although the few references in the literature remain inconclusive, taken together with our presented data we hypothesize that the lack of AQP1 expression in the myenteric has a direct influence on the muscle layer and might inflict intestinal muscle function, while the lack of AQP1 in the submucosal plexus as well as in the mucosa influences the water homeostasis in the colon and with this the stool microbiome.

How the water channel function, the ion channel function, or a possible gas channel function of AQP1 protein impact these processes in detail remains the focus of further research.

Our findings also hint that the ability of the mice to concentrate stool might be compromised, indicating once more an important role of AQP1 in water homeostasis by its water channel function. Our data furthermore showed that the loss of AQP1 expression was associated with a changed composition of microbiota in the stool.

Heat production as measured by isothermal microcalorimetry is a measure of the overall metabolic activity, in this case, of bacteria. Our measurements suggested a different bacterial composition of WT and AQP1-KO stool, which could be confirmed by detailed microbiome analysis. Detecting differences in the composition of stool bacteria by measuring heat production, as was proven by the microbiome analysis, could provide the base for a fast and fairly simple differentiation of bacterial composition of stool samples in the future.

Alpha diversity of WT and AQP-KO mice do not seem to differ much. Since both indices (Shannon- and Simpson-index) consider richness and abundance of the samples, results must be interpreted with caution. Beta-diversity in our experiment showed a clear shift of the phyla. Earlier studies have already shown that the phylum of bacteroidetes and firmicutes are the two largest phyla in the gut of the mice as well as of humans [41,62]. In all our mice these two phyla represent the largest category. However, loss of AQP1 leads to a slightly lower amount of bacteroidetes and firmicutes but a higher amount of the rest of the phyla especially deferribacteres, proteobacteria, and verrucomicrobia. In inflammatory diseases the gut microbiome in mice differs from the one of healthy mice [62]. For example, it has been found that deferribacteres and verrucomicrobia increase in chronic enteritis [62]. Recent studies have also suggested the involvement of the gut microbiome in neurodegenerative processes such as Parkinson’s disease or methylmercury poisoning [42,63]. According to Benech et al. tryptophane metabolites activate the gut movement and these metabolites are produced by the microbiome [43]. A microbiome shift is also observed in Hirschsprung’s disease (HD) and Hirschsprung’s Disease Associated Enterocolitis (HAEC), leading to severe colitis [64]. In HD ganglia are missing in the colon leading to a lack of colon function including muscular and mucosal function. Furthermore, we have previously described an increase in AQP1 expression in acute appendicitis [25]. Further research is necessary to investigate the correlation of different functions of AQP1 expressed in its distinct locations in the gastrointestinal tract with the microbiome and metabolic measurements of stool samples from each region to achieve higher spacial resolution.

## 4. Materials and Methods

### 4.1. Tissue Preparation

Ten mice were bred from a C57BL/6 background (5 WT AQP1^+/+^, 4 KO AQP1^−/−^ and 1 HT AQP1^−/+^) and the intestines provided by from the Department of Physiology of the Hannover Medical School (Hannover, Germany). The AQP1-KO mice had been generated from breeding pairs of heterozygous AQP^−/+^ mice kindly provided by Dr. Alan S. Verkman (San Francisco, CA, USA; Ma et al., 1998). Animals were anaesthetized and sacrificed in accordance with the German Tierschutzgesetz §4 2015/222. Organs, specifically the esophagus, stomach, duodenum, jejunum, ileum, cecum, and colon were immediately removed. These organs were separately snap frozen and cryo-conserved. Each organ was embedded in OCT (TissueTek; Thermofisher Scientific Inc., Waltham, MA, USA) according to a modification of the “Swiss role” described by Meier-Ruge [65], with exception of the esophagus, which was embedded as a tube for cryo-cutting. The samples were cut with the cryotome (Leica CM1950) into 7 µm slices, fixed on a microscope slide and stored at minus 20 °C.

### 4.2. Heterozygous Mouse

One of the five originally as AQP1-KO classified mice was reclassified as heterozygous (AQP1^−/+^) when IHC staining was positive for AQP1, and heterozygous DNA expression was confirmed. Results of this mouse were excluded from all analysis except for the pooled stool analysis.

### 4.3. Immunohistochemistry and Immunofluorescence

The slices were stained by routine H.E. staining as well as immunohistochemically stained for AQP1 with the Cell and Tissue staining Rabbit Kit HRP-AEC System (R&D Systems, Minneapolis, MN, USA). The kit was used according to the R&D System’s protocol, and a rabbit primary antibody against AQP1 (Sigma-Aldrich, Merck, Millipore, Burlington, MA, USA) was used at a dilution of 1:400. The samples were than counterstained with hematoxylin (Spitalpharmazie USB, Basel, Switzerland), mounted with aquatex (Merck KGaA, Darmstadt, Germany) and covered up with a Cover-Slip. Every staining was accompanied with a negative control using antibody diluent (DAKO, Glostrup, Denmark).

Immunofluorescence staining was performed as a triple staining with antibodies against AQP1, calretinin, and S100B. The staining was performed according to a standardized protocol, using the rabbit anti-AQP1 antibody (Merck Millipore, Darmstadt, Germany) at a dilution of 1:400, the chicken anti-calretinin antibody (Synaptic Systems, Göttingen, Germany) at a dilution of 1:200 and the guinea pig anti-S100B antibody (Synaptic Systems, Göttingen, Germany) at a dilution of 1:400. As secondary antibodies, anti-rabbit A488 (Invitrogen, Carlsbad, CA, USA), anti-chicken A647 (Invitrogen, Carlsbad, CA, USA), and anti-guinea pig A555 (Invitrogen, Carlsbad, CA, USA) were used at a dilution of 1:2000. Tissue slices were mounted with DAPI (Life Technologies, Thermo Fischer Scientific Inc., Waltham, MA, USA). For every sample an additional negative control without the primary antibodies, was carried out.

### 4.4. Staining Analysis

The immunohistochemical and the immunofluorescence stainings were analyzed using an Olympus BX63 microscope with the accompanying CellSens Software (Olympus). To evaluate the intensity of AQP1 expression in the immunohistochemical staining two independent examiners scored representative specimen sections of each mouse and each organ. A scoring system with a range from 0 to 3 (0—negative for AQP1, 3—most intense AQP1 expression) was applied. Results of both examiners were correlated. In the few cases of incongruity, the sections were reexamined, and a joined scoring was reached. Furthermore, the immunohistochemical pictures were analyzed with ImageJ-win32 to measure the color intensity of AQP1 for each picture in the wildtype mice. For the measurement a threshold was set by HSB 195–255/30–255/0–200 as a reference to our immunohistochemical staining and the color intensity was measured with this border. The results were depicted as the sum of pixels in each picture according to these settings.

Furthermore, the thickness of the anatomical layers (entire intestinal wall, mucosa, and muscular layer) of the different parts of the intestine was measured using ImageJ-win64. All intestinal samples of AQP-KO mice (*n* = 4) and WT mice (*n* = 5) were measured. A total of 4–6 measurements were taken from each intestinal segment per mouse.

### 4.5. Wet-to-Dry Ratio of Intestinal Segments

Intestinal samples were cleaned of intraluminal contents and wet wight was measured. Samples were then dried in an 80 °C oven for 48 h, when dry weight was constant. Then dry weight was measured. The wet-to-dry ratio was then determined by calculating: (wet weight − dry weight)/dry weight.

### 4.6. RNA Isolation/cDNA Synthesis/qPCR

Tissue slices of 50µm were shredded and lysed in Buffer RLT Plus (QIAGEN). RNA isolation was subsequently performed using the RNeasy Plus Mini Kit (QIAGEN, Cat. No. 74134) according to the manual. RNA was eluted in 30 µL or 14 µL nuclease-free water, respectively. RNA concentration was determined using a Colibri Microvolume Spectrometer (BioConcept AG). cDNA synthesis was performed using the GoScript™ Reverse Transcription System (Promega, Cat. No. A5000) from up to 200 ng RNA per reaction. A Biometra T-Personal Thermal Cycler was used. Quantitative PCR was performed using the FastStart Universal SYBR Green Master (Rox) (Roche, Cat. No. 4913850001). AQP1, HIF-1α, and PGK1 specific primers (Microsynth, Balgach, Switzerland) were used for the amplification of cDNA. Per reaction, 5 µL Master Mix was mixed with primers forward and reversed (0.5 µM), 1 µL of cDNA template or water in NTC, and water to a final volume of 10 µL. PCR reactions were performed in triplicates in MicroAmp™ Optical 384-Well Reaction Plates (Applied Biosystems, Cat. No. 4309849) in a ViiA 7 Real-Time PCR System (Applied Biosystems) using the associated software. Data were analyzed using the 2^−ΔCT^ method × 1000.

### 4.7. Isothermal Microcalorimetry

For microcalorimetric measurements a differential nanocalorimeter (TAM III nano, Waters/TA Delaware, USA) was used. Stool samples were weighted to ensure that samples of similar weight were processed and to allow for further standardization. They were transferred into 3 mL calorimetric glass vials. Vials were then sealed and inserted according to the manufacturer’s instructions. Sterile glass vials filled with the same weight of sterile saline solution served as reference samples, acting as inert thermal references. Following thermal equilibration, measurements were recorded with the thermostat set at 37 °C. The microcalorimeter data were sampled at a frequency of 1 data point per second and further resampled to obtain an effective sampling rate of 1 data point every 300 s over >250 h (i.e., until the metabolic heat signal returned to baseline). Data were stored by the TAM assistant software and exported as a CSV file that could be edited in commonly used spreadsheet software. As we did not perform this experiment with biological replicates, statistical analysis was not possible.

### 4.8. Dry Weight Measurement

Before removal of the organ, stools were sampled from all individually caged 10 mice immediately after defecation and put into weighing bottles in Hannover. After determining the wet weights, the stool samples were dried in a drying chamber at 105 °C for 24 h and weighed again to obtain the stools’ dry weight.

### 4.9. Graphs and Statistical Analysis

Data were analyzed using Microsoft Excel, R [66] and Graph Prism 9 Software. Data was tested for normal distribution with the Shapiro–Wilk test. Comparison of group means was carried out using the *t*-test if data were normally distributed and the Mann–Whitney test if not. *p*-values less or equal to 0.05 were considered statistically significant. Graphs were created utilizing Graph Prism 9 software or R, using the R-packages “ggplot2” [67] and “ggpubr” [68].

### 4.10. Microbiome Analysis

Bacterial RNA was isolated from the stool samples of each mouse separately. RNA was analyzed though a service by Microsynth AG Balgach Switzerland according to their protocols. Different bacterial RNA were characterized and their relative abundances measured by the company.

The biodiversity in the gut microbiota samples was assessed using the Shannon and the Simpson index [69]. The compositional dissimilarity among the gut microbiota samples was assessed using the Bray–Curtis statistic [69]. The significance of the dissimilarities found was assessed using a non-parametric multivariate analysis of variance [70]. All calculations were carried out using R and the R-packages “phyloseq” [71] and “vegan” [72].

## 5. Conclusions

AQP1 is found in a specific pattern in the serosa, the mucosa, and the enteric nervous system throughout the gastrointestinal tract. Although AQP-KO mice retained gastrointestinal function, distinct changes regarding the anatomy of the intestinal wall were observed. Loss of AQP1 might interfere with the ability of the mice to concentrate their stool and is associated with a significantly different bacterial expression pattern of the stool microbiome.

## Data Availability

All data are depicted in the manuscript and raw data can additionally be requested from the corresponding author.

## Supplementary Materials

The following supporting information can be downloaded at: https://www.mdpi.com/article/10.3390/ijms24043616/s1.
