# Supplementary material for: AQP1 in the Gastrointestinal Tract of Mice: Expression Pattern and Impact of AQP1 Knockout on Colonic Function
Source: Int J Mol Sci. 2023 Feb 10;24(4):3616. doi: 10.3390/ijms24043616 (PMC9959819; doi:10.3390/ijms24043616)
Supplement: Supplementary file 1 [file ijms-24-03616-s001.zip › ijms-2082562-supplementary.pdf]

International Journal of *Molecular Sciences*

MDPI

Supplementary Materials

AQP1 in the Gastrointestinal Tract of Mice: Expression Pattern and Impact of AQP1 Knockout on Colonic Function

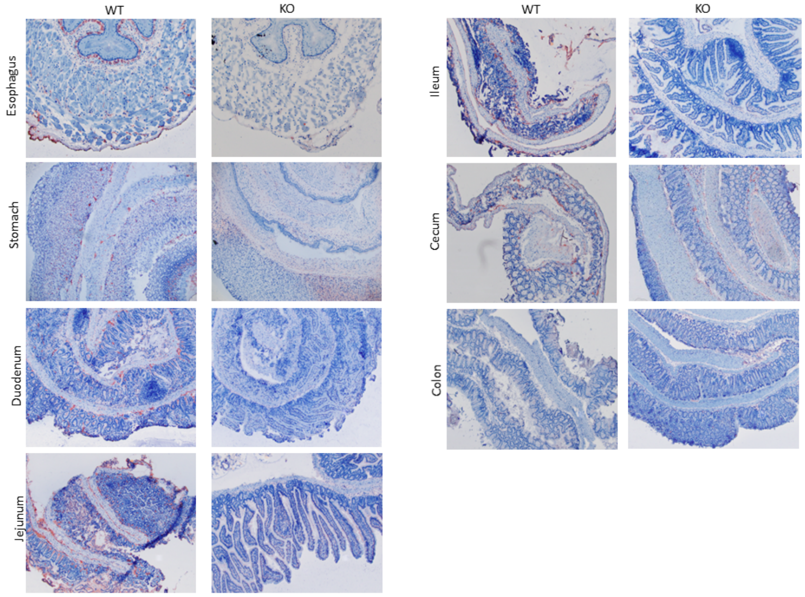

Figure S1. AQP1 expression in different parts of the gastrointestinal tract (WT and AQP1-KO).

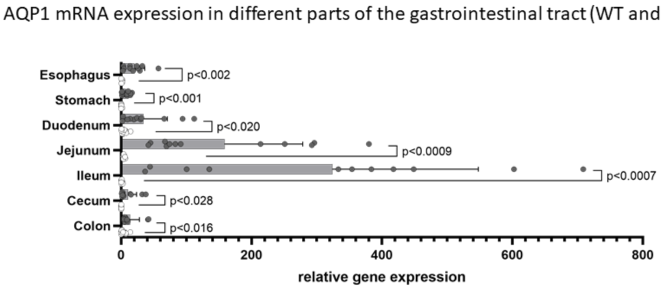

Figure S2. AQP1 mRNA expression in different parts of the gastrointestinal tract (WT and AQP1-KO);.

Table S1. Intestinal wall WT and AQP1-KO mean, standard deviation (SD) and number of measurements (N). All measurements in µm. .

	Intestinal wall WT			Intestinal wall AQP1-KO			Difference
	Mean	SD	N	Mean	SD	N	p-value
**Esophagus**	280.82	87.51	30	240.97	94.35	24	*0.128*
**Stomach**	415.19	114.94	30	609.07	125.73	24	*<0.0001*
**Duodenum**	229.09	96.82	24	261.65	94.87	24	*0.245*
**Jejunum**	325.57	129.79	27	620.67	213.53	24	*<0.000001*
**Ileum**	313.30	143.58	27	417.34	67.69	24	*0.002*
**Cecum**	255.53	63.55	30	294.59	72.22	24	*0.325*
**Colon**	481.29	108.86	30	377.01	79.79	24	*0.006*

Table S2. Muscular layer WT and AQP1-KO mean, standard deviation (SD) and number of measurements (N). All measurements in μm.

	Muscular layer WT			Muscular layer AQP1-KO			Difference
	**Mean**	**SD**	**N**	**Mean**	**SD**	**N**	**p-value**
**Esophagus**	211.08	60.19	30	173.94	68.24	24	*0.039*
**Stomach**	132.01	64.76	30	153.98	85.75	24	*0.190*
**Duodenum**	54.89	15.97	24	71.96	28.89	24	*0.016*
**Jejunum**	61.34	21.85	27	78.70	36.18	24	*0.048*
**Ileum**	75.43	26.17	30	87.40	19.62	24	*0.069*
**Cecum**	72.58	23.81	29	99.03	36.83	24	*0.004*
**Colon**	178.88	58.72	30	136.84	49.00	24	*0.007*

Table S3. Mucosal layer WT and AQP1-KO mean, standard deviation (SD) and number of measurements (N). All measurements in μm.

	Mucosal layer WT			Musosal layer AQP1-KO			Difference
	**Mean**	**SD**	**N**	**Mean**	**SD**	**N**	**p-value**
**Esophagus**	69.74	31.88	30	67.02	28.43	24	*0.742*
**Stomach**	283.18	108.25	30	455.09	53.91	24	*<0.0001*
**Duodenum**	174.21	84.20	24	189.69	75.93	24	*0.507*
**Jejunum**	264.23	116.99	27	541.97	201.89	24	*<0.000001*
**Ileum**	236.86	129.92	27	329.95	63.98	24	*0.002*
**Cecum**	178.90	48.66	30	195.56	57.30	24	*0.415*
**Colon**	302.41	74.26	30	240.17	51.65	24	*0.0007*
